# Dr. Henry Baethig

**Published:** 1906-08

**Authors:** 


Dr. Henry Baethig, of Buffalo, died at his home, No. 350 Pennsylvania Street, July 14, 1906, from a sudden attack of heart failure, aged 56 years. Dr. Baethig was a native of Nuremberg, Germany, and came to Buffalo with his parents when he was an infant. He received his preliminaries in the public schools of this city and took his doctorate degree at the Hahnemann Institute at Philadelphia. He has practised his profession in this city since graduation and of late years has been regarded as one of our leading homeopathic physicians.

